# Supplementary material for: First fully-automated AI/ML virtual screening cascade implemented at a drug discovery centre in Africa
Source: Nat Commun. 2023 Sep 15;14:5736. doi: 10.1038/s41467-023-41512-2 (PMC10504240; doi:10.1038/s41467-023-41512-2)
Supplement: Supplementary file 1 — Supplementary Information [file 41467_2023_41512_MOESM1_ESM.pdf]

Supplementary Information

First fully-automated AI/ML virtual screening cascade implemented at a drug discovery centre in Africa

Gemma Turon*[,1], Jason Hlozek*[,2], John G. Woodland[2,3], Ankur Kumar[1], Kelly Chibale[2,3,c], Miquel Duran-Frigola[1,c]

[1] Ersilia Open Source Initiative, Cambridge, United Kingdom

[2] Department of Chemistry and Holistic Drug Discovery and Development (H3D) Centre, University of Cape Town, South Africa

[3] South African Medical Research Council Drug Discovery and Development Research Unit, Institute of Infectious Disease and Molecular Medicine, University of Cape Town, South Africa

[c] Corresponding authors:

Kelly Chibale: kelly.chibale@uct.ac.za
Miquel Duran-Frigola: miquel@ersilia.io

* These authors contributed equally to this work.

Assay	Description	Molecules	% Actives	Cut-off
P. falciparum NF54 IC_{50}	Half-maximal inhibitory concentration of *P. falciparum* NF54 strain cultured at 5% parasitaemia in human blood	3,289	21.19	0.1 µM
P. falciparum K1 IC_{50}	Half-maximal inhibitory concentration of *P. falciparum* K1 strain cultured at 5% parasitaemia in human blood	1,425	23.58	0.1 µM
M. tuberculosis H37Rv MIC_{90}	Minimal inhibitory concentration 90% of *M. tuberculosis* H37Rv strain cultured in glucose-Tween80 media	3,244	16.55	5 µM
CHO IC_{50}	Half-maximal inhibitory concentration of CHO cell growth	2,029	16.36	10 µM
HepG2 IC_{50}	Half-maximal inhibitory concentration of HepG2 cell growth	1,457	40.22	10 µM
CL_{int} Human	Human microsomal clearance *in vitro*	1,430	51.12	11.6 µg/min/mg
CL_{int} Mouse	Mouse microsomal clearance *in vitro*	1,165	61.12	11.6 µg/min/mg
CL_{int} Rat	Rat microsomal clearance *in vitro*	1,202	58.99	11.6 µg/min/mg
Aqueous solubility	Solubility at pH 7.4	3,227	55.78	90 µM
Aqueous solubility	Solubility at pH 6.5	2,326	47.21	90 µM
Caco-2	Passive membrane permeability	134	62.69	10e-6 cm/s
CYP2C9 IC_{50}	Half-maximal inhibitory concentration of cytochrome CYP2C9 (from literature)	16,379	34.74	10 µM
CYP2C19 IC_{50}	Half-maximal inhibitory concentration of cytochrome CYP2C19 (from literature)	15,551	43.36	10 µM
CYP2D6 IC_{50}	Half-maximal inhibitory concentration of cytochrome CYP2D6 (from literature)	17,812	24.38	10 µM
CYP3A4 IC_{50}	Half-maximal inhibitory concentration of cytochrome CUP3A4 (from literature)	21,810	41.94	10 µM
hERG IC_{50}	Blockade of the human ether-a-go-go-related gene potassium channel (from literature)	12,620	52.64	10 µM

Supplementary Table 1. Bioassay descriptions with dataset sizes and activity cut-offs for the classification models.

Dataset	Metric	Score	Position*
Bioavailability_Ma	AUROC	0.745 ± 0.012	2nd
HIA_Hou	AUROC	0.987 ± 0.003	2nd
Pgp_Broccatelli	AUROC	0.942 ± 0.001	1st
BBB_Martins	AUROC	0.933 ± 0.003	4th
CYP2C9_Veith	AUPRC	0.787 ± 0.004	3rd
CYP2D6_Veith	AUPRC	0.717 ± 0.008	3rd
CYP3A4_Veith	AUPRC	0.87 ± 0.003	4th
CYP2C9_Substrate_CarbonMangels	AUPRC	0.439 ± 0.012	2nd
CYP2D6_Substrate_CarbonMangels	AUPRC	0.724 ± 0.008	2nd
CYP3A4_Substrate_CarbonMangels	AUPRC	0.661 ± 0.008	2nd
hERG	AUROC	0.861 ± 0.007	2nd
AMES	AUROC	0.853 ± 0.007	2nd
DILI	AUROC	0.933 ± 0.005	1st

Supplementary Table 2. ZairaChem model performance on the Therapeutics Data Commons ADMET Leaderboard. The score is the mean ± standard deviation of the indicated metric on five-fold cross validation. *Position refers to expected position in each leaderboard at time of submission.

Assay	AUROC	Standard deviation
P. falciparum NF54 IC_{50}	0.902	0.009
P. falciparum K1 IC_{50}	0.889	0.03
M. tuberculosis H37Rv MIC_{90}	0.903	0.007
CHO IC_{50}	0.871	0.01
HepG2 IC_{50}	0.973	0.006
CL_{int} Human	0.816	0.03
CL_{int} Mouse	0.802	0.033
CL_{int} Rat	0.802	0.023
Aqueous solubility (pH=7.4)	0.893	0.005
Aqueous solubility (pH=6.5)	0.884	0.014
Caco-2	0.941	0.033
*CYP2C9 IC_{50}	0.803	0.04
*CYP2C19 IC_{50}	0.618	0.037
*CYP2D6 IC_{50}	0.666	0.027
*CYP3A4 IC_{50}	0.793	0.051
*hERG IC_{50}	0.852	0.014

Supplementary Table 3. Model performances (area under the ROC curve (AUROC) and standard deviation). Models have been evaluated on 20% of the total data and five-fold cross-validated. All splits have been stratified by actives/inactives. *Models developed with external data and models from the literature have been validated on 75% of the internal data at each fold. Source data are provided as a Source Data file.

Assay	Hit rate improvement in active compounds in the top 50 (%)	Hit rate improvement in inactive compounds in the bottom 50 (%)
P. falciparum NF54 IC_{50}	70	22
P. falciparum K1 IC_{50}	58	24
M. tuberculosis H37Rv MIC_{90}	68	16
CHO IC_{50}	44	18
HepG2 IC_{50}	58	42
CL_{int} Human	42	32
CL_{int} Mouse	26	40
CL_{int} Rat	28	36
Aqueous solubility (pH=7.4)	46	54
Aqueous solubility (pH=6.5)	52	44
Caco-2	Insufficient number of test molecules	Insufficient number of test molecules
*CYP2C9 IC_{50}	20	20
*CYP2C19 IC_{50}	0	20
*CYP2D6 IC_{50}	20	20
*CYP3A4 IC_{50}	30	20
hERG IC_{50}	28	46

Supplementary Table 4. Hit enrichments in the top 50 and bottom 50 molecules ranked according to the model score (probability of 1). *Cytochrome test sets feature < 60 molecules in total, therefore hit enrichments are calculated over the top 10 and bottom 10 molecules. Source data are provided as a Source Data file.

Cut-off 0.1	Precision	Recall	F1	TP	TN	FP	FN
Napthyridines *Pf* NF54	0.183	1.0	0.309	2	76	0	17
Napthyridines Solubility (pH 6.5)	0.407	1.0	0.578	0	54	0	37
Pyrazoles *Mtb*	0.577	0.872	0.695	8	30	6	41
Pyrazoles Solubility (pH 7.4)	0.718	1.0	0.836	0	22	0	56

Cut-off 0.3	Precision	Recall	F1	TP	TN	FP	FN
Napthyridines *Pf* NF54	0.333	0.529	0.409	60	18	8	9
Napthyridines Solubility (pH 6.5)	0.648	0.946	0.769	35	19	2	35
Pyrazoles *Mtb*	0.778	0.298	0.431	34	4	33	14
Pyrazoles Solubility (pH 7.4)	0.727	1.0	0.842	1	21	0	56

Cut-off 0.5	Precision	Recall	F1	TP	TN	FP	FN
Napthyridines *Pf* NF54	0.667	0.118	0.200	77	1	15	2
Napthyridines Solubility (pH 6.5)	0.765	0.703	0.732	46	8	11	26
Pyrazoles *Mtb*	0.0	0.0	0.0	38	0	47	0
Pyrazoles Solubility (pH 7.4)	0.814	0.625	0.707	14	8	21	35

Supplementary Table 5. Model performance metrics for prospective compounds in two chemical series where model probability scores have been classified as “active” according to increasingly stringent thresholds. Cut-offs represent different cut-off choices, from relatively permissive (0.1) to more stringent (0.5). Metrics are reported for precision, recall, f1-score (F1), true positives (TP), true negatives (TN), false positives (FP), and false negatives (FN) at each cut-off. Source data are provided as a Source Data file.

Assay	AUROC	Standard deviation	% of performance maintained
P. falciparum NF54 IC_{50}	0.87	0.02	96
P. falciparum K1 IC_{50}	0.87	0.02	96.51
M. tuberculosis H37Rv MIC_{90}	0.86	0.02	95.42
CHO IC_{50}	0.83	0.03	95.2
HepG2 IC_{50}	0.95	0.01	97.73
CL_{int} Human	0.79	0.02	97.26
CL_{int} Mouse	0.79	0.03	98.2
CL_{int} Rat	0.77	0.03	95.48
Aqueous solubility (pH=7.4)	0.86	0.02	96.39
Aqueous solubility (pH=6.5)	0.85	0.01	96.69
Caco-2	0.92	0.07	98.26
*CYP2C9 IC_{50}	0.79	0.05	98.8
*CYP2C19 IC_{50}	0.6	0.05	97.55
*CYP2D6 IC_{50}	0.65	0.05	96.96
*CYP3A4 IC_{50}	0.82	0.03	103.62

Supplementary Table 6. Model performances (area under the ROC curve (AUROC) and standard deviation) for the light–weight models built with H3D data and provided through the deployed app. Models have been evaluated on 20% of the total data and five-fold cross-validated. *Models developed with external data have been validated on 75% of the internal data at each fold. Source data are provided as a Source Data file.

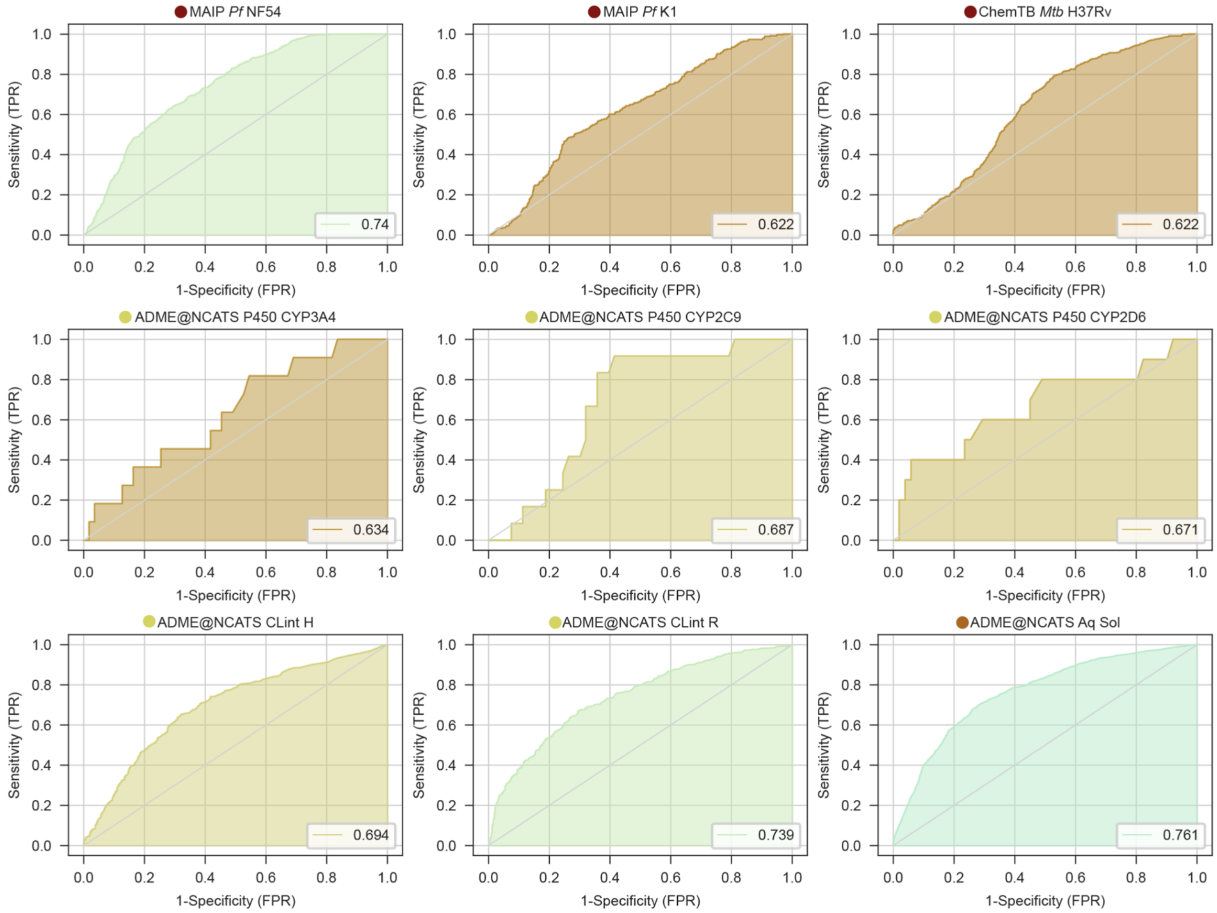

Supplementary Figure 1. ROC curves with corresponding AUROC scores of the performance of third-party models on H3D data. Source data are provided as a Source Data file.

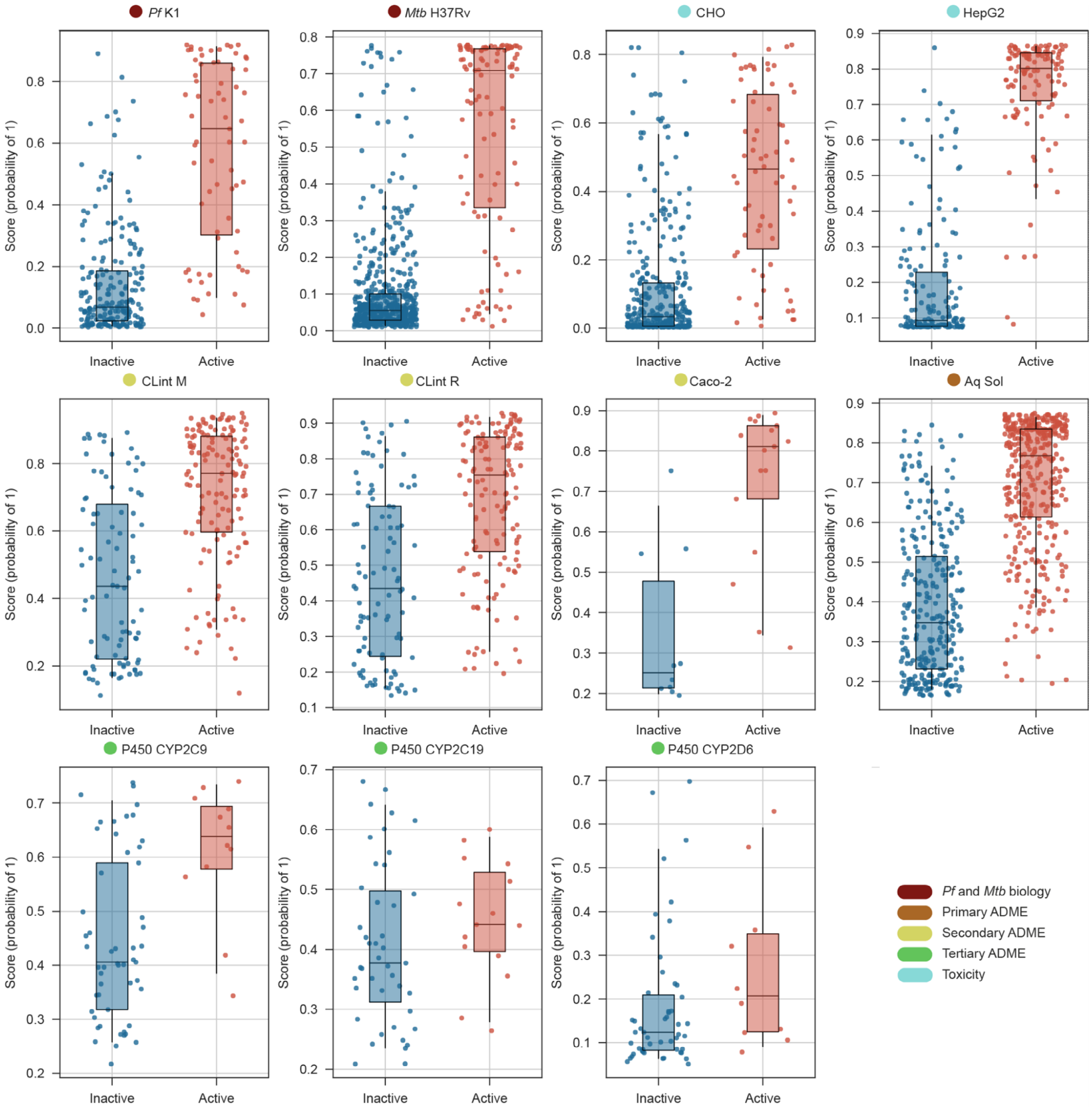

Supplementary Figure 2. Scores (probability of 1) obtained by ZairaChem classification models on test data (20% of total dataset). Blue indicates true inactive molecules and red represents true active molecules. Only one representative fold is shown. (n active/inactive: *Pf* K1: 67/218, *Mtb* H37Rv: 107/524, CHO: 66/340, HepG2: 117/175, CLintM: 142/91, CLintR: 142/99, Caco-2: 17/10, Aq Sol: 139/102, CYP2C9: 9/39,CYP2C19: 11/32, CYP2D6: 7/38) *Models developed with external data and models from the literature have been validated on 75% of the internal data at each fold. Boxes indicate the median (central line), Q1 (upper bound) and Q3 (lower bound) and whiskers extend to the data points within up to 1.5 times in the interquartile range. Source data are provided as a Source Data file.

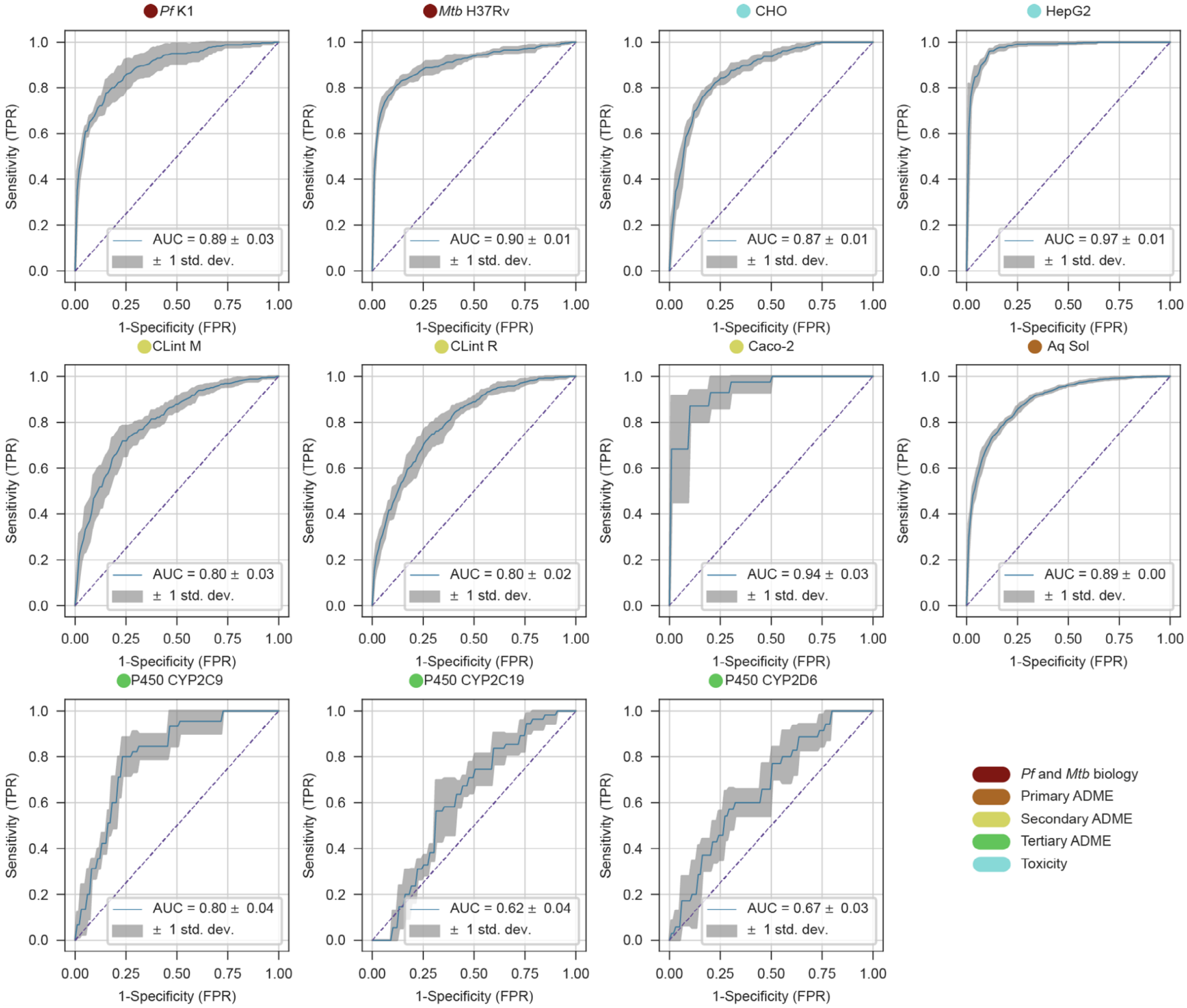

Supplementary Figure 3. ROC curves of 11 ZairaChem models and associated AUC values ± standard deviations (std. dev.). Models have been five-fold cross-validated using a random stratified 80-20 split. *Models developed with external data and models from the literature have been validated on 75% of the internal data at each fold. Blue lines represent the mean of the five folds. Source data are provided as a Source Data file.

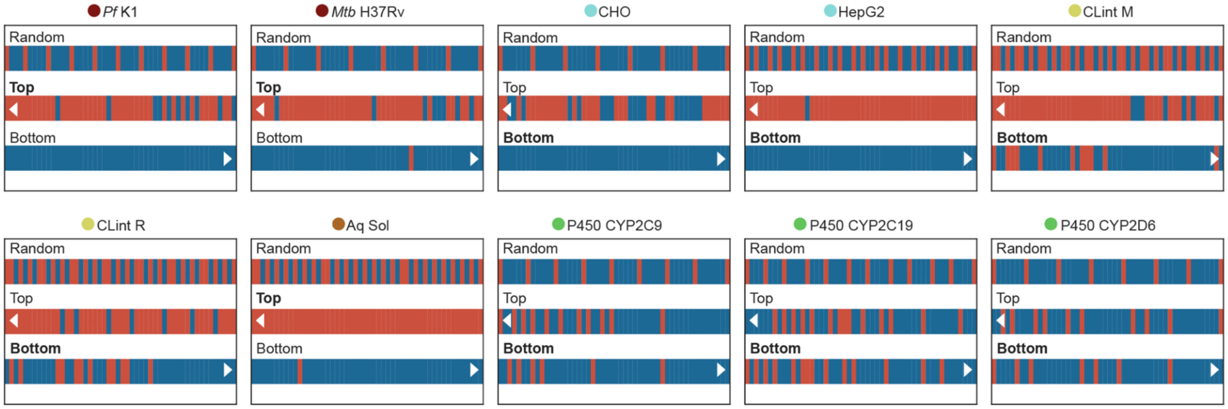

Supplementary Figure 4. Comparison of hit rates for randomly selected molecules (first row) vs molecules ranked according to the model score (probability of 1, second and third rows) for ten assays corresponding to activity against *Pf* and *Mtb*, cytotoxicity, intrinsic microsomal clearance in mice (M) and rats (R), aqueous solubility, and inhibition of CYP enzymes. The top 50 and bottom 50 molecules are depicted, showing a hit enrichment of true active compounds (red) in the highest-ranked positions and an enrichment of true inactive compounds (blue) in the lowest-ranked compounds. Red and blue arrows, respectively, represent the desired experimental outcome for molecule progression in the cascade. Source data are provided as a Source Data file.

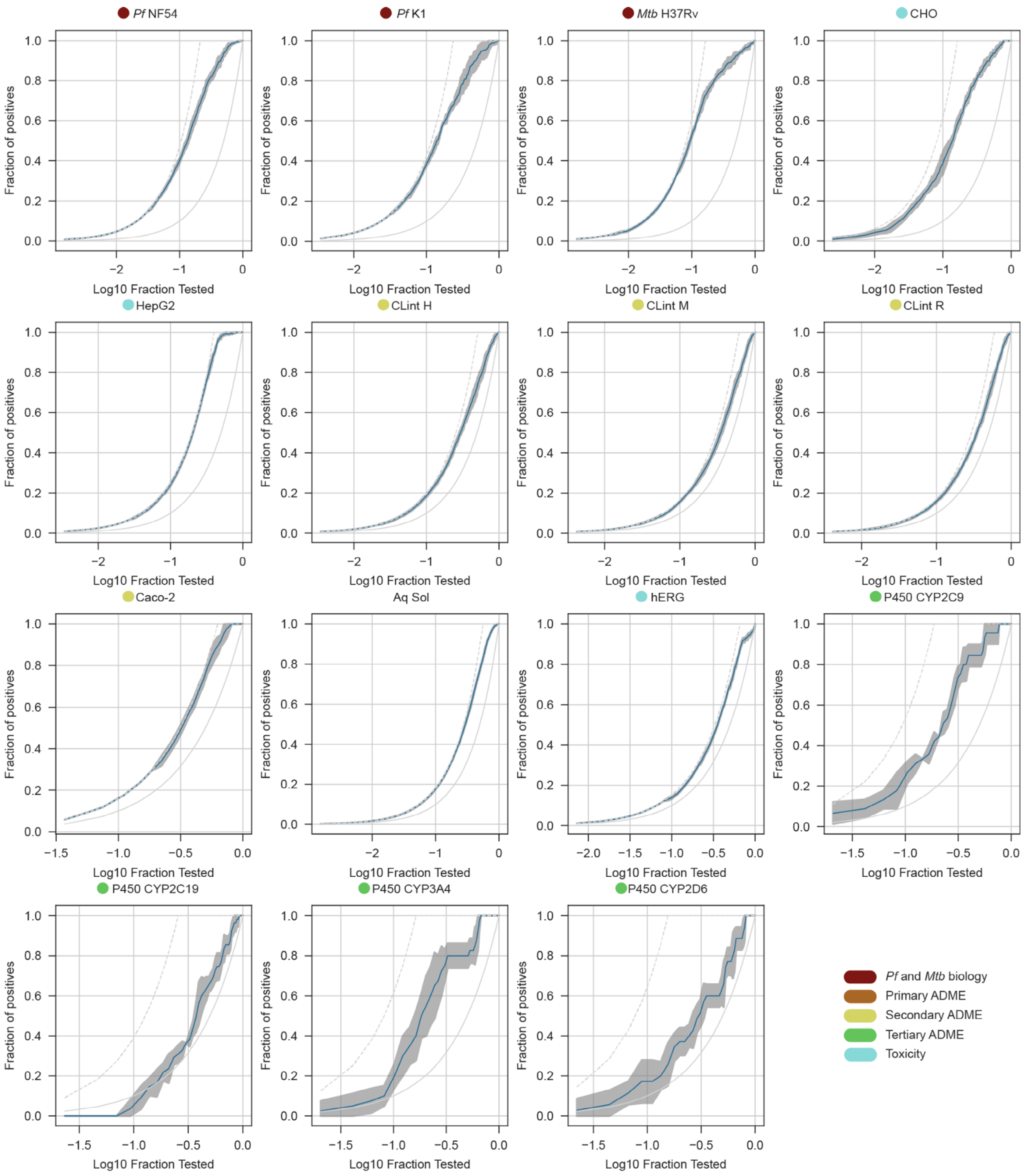

Supplementary Figure 5. Hit enrichment curves for the 15 models deployed as a virtual screening cascade. Blue lines represent the mean hit enrichment of the five-fold model cross-validations ± standard deviation (grey areas). Left and right grey lines represent the ideal situation (all actives are identified first) and the random situation (all molecules in the test set must be screened to identify all the active molecules), respectively. Source data are provided as a Source Data file.

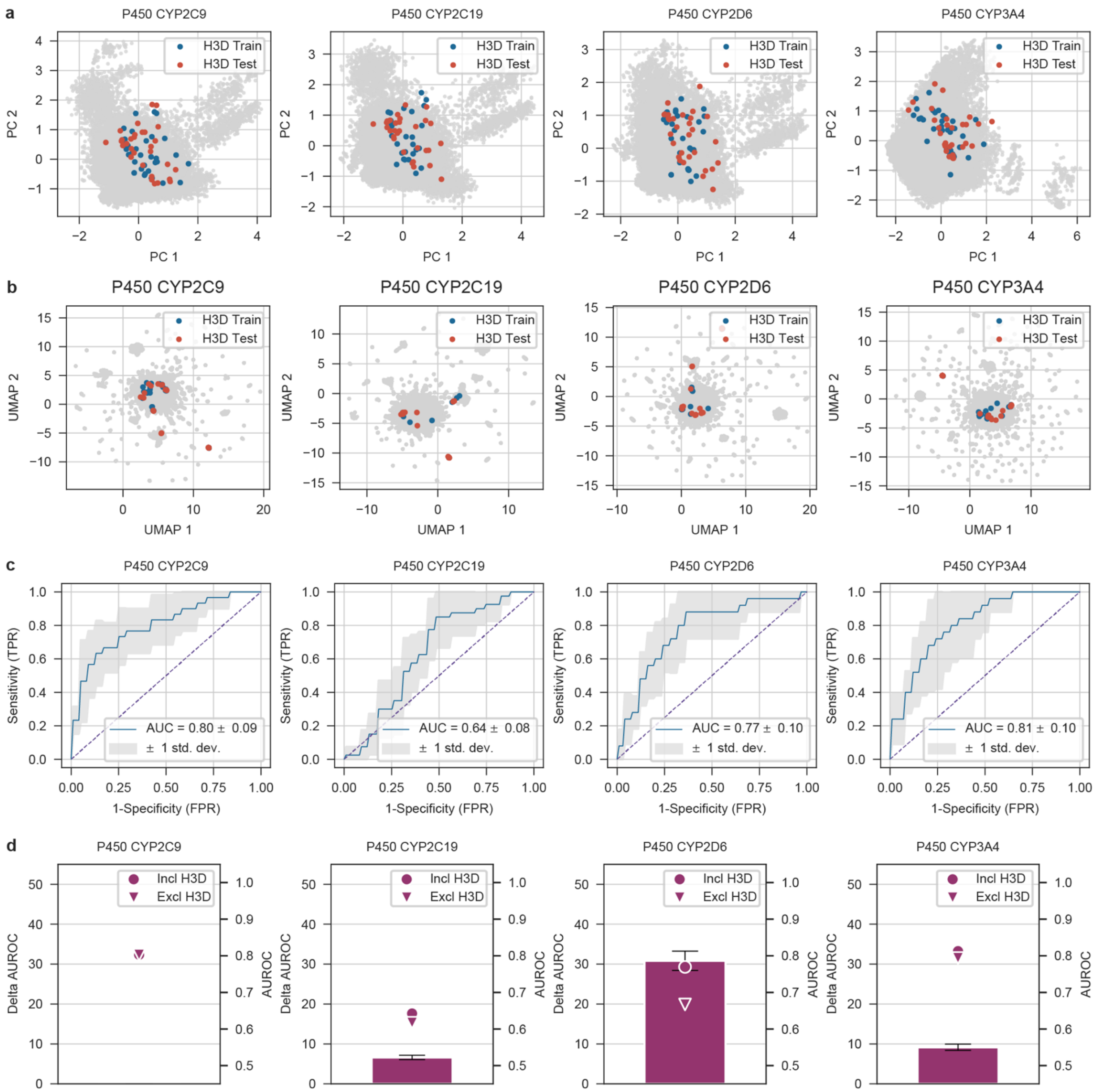

Supplementary Figure 6. ZairaChem models for CYP P450 inhibition trained on external datasets+50% of internal H3D data points (approximately 30 molecules). The distribution of chemical space for each cytochrome dataset is depicted as two-dimensional projections with (**a**) principal component analysis and (**b**) uniform manifold approximation and projection. These projections correspond to the first data fold from a five-fold cross validation, with public data (grey), H3D data included in the training set (blue) and H3D data used for the test set (red). (**c**) The mean ROC curves from the five-fold cross validation with standard deviations. (**d**) Percentage change in AUROC score (left y-axis) towards a perfect model (AUROC = 1) when adding internal data to the external training data (see 'AUROC percentage change' in Materials and Methods for analogous calculation). The right y-axis shows the actual AUROC values for the models before (downward triangle) and after (circle) adding internal data. Error bars indicate +/- standard

deviation (n = 5). Source data are provided as a Source Data file.

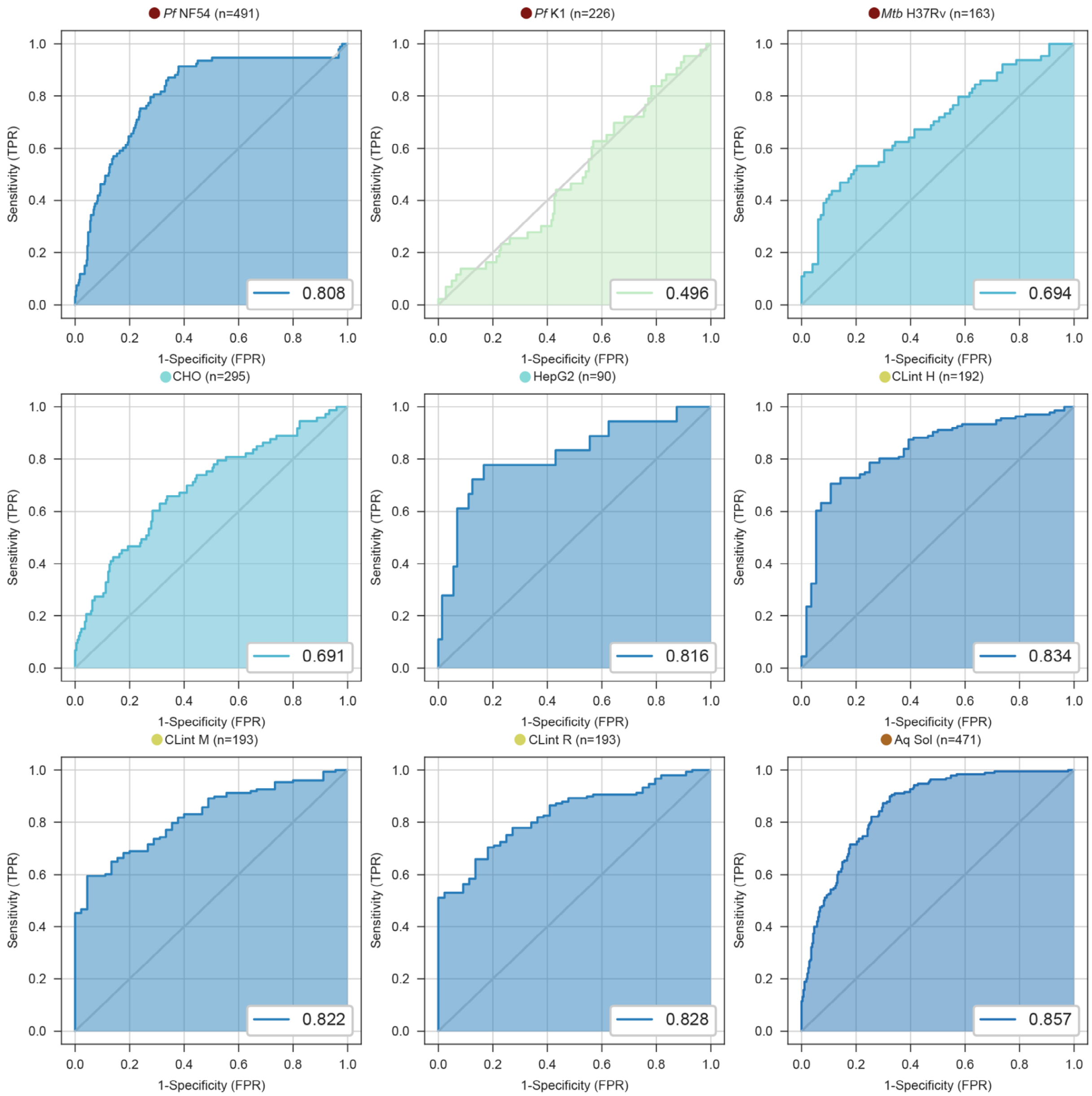

Supplementary Figure 7. ROC curves with corresponding AUROC scores for prospective data produced at the H3D Centre during 2022 for the ZairaChem virtual screening cascade models trained on internal data; activity against *Pf* and *Mtb*, solubility, cytotoxicity, and intrinsic clearance in mouse (M), rat (R) and human (H) liver microsomes. Source data are provided as a Source Data file.
