# Supplementary material for: First fully-automated AI/ML virtual screening cascade implemented at a drug discovery centre in Africa
Source: Nat Commun. 2023 Sep 15;14:5736. doi: 10.1038/s41467-023-41512-2 (PMC10504240; doi:10.1038/s41467-023-41512-2)
Supplement: Supplementary file 3 — Reporting Summary [file 41467_2023_41512_MOESM3_ESM.pdf]

nature portfolio

Corresponding author(s): Prof. Kelly Chibale
Dr. Miquel Duran-Frigola

Last updated by author(s): 2023/06/23

Reporting Summary

Nature Portfolio wishes to improve the reproducibility of the work that we publish. This form provides structure for consistency and transparency in reporting. For further information on Nature Portfolio policies, see our Editorial Policies and the Editorial Policy Checklist.

Statistics

For all statistical analyses, confirm that the following items are present in the figure legend, table legend, main text, or Methods section.

n/a	Confirmed	
☐	☒	The exact sample size (*n*) for each experimental group/condition, given as a discrete number and unit of measurement
☐	☒	A statement on whether measurements were taken from distinct samples or whether the same sample was measured repeatedly
☒	☐	The statistical test(s) used AND whether they are one- or two-sided<br>*Only common tests should be described solely by name; describe more complex techniques in the Methods section.*
☐	☒	A description of all covariates tested
☐	☒	A description of any assumptions or corrections, such as tests of normality and adjustment for multiple comparisons
☐	☒	A full description of the statistical parameters including central tendency (e.g. means) or other basic estimates (e.g. regression coefficient) AND variation (e.g. standard deviation) or associated estimates of uncertainty (e.g. confidence intervals)
☒	☐	For null hypothesis testing, the test statistic (e.g. *F*, *t*, *r*) with confidence intervals, effect sizes, degrees of freedom and *P* value noted<br>*Give P values as exact values whenever suitable.*
☒	☐	For Bayesian analysis, information on the choice of priors and Markov chain Monte Carlo settings
☐	☒	For hierarchical and complex designs, identification of the appropriate level for tests and full reporting of outcomes
☒	☐	Estimates of effect sizes (e.g. Cohen's *d*, Pearson's *r*), indicating how they were calculated

Our web collection on statistics for biologists contains articles on many of the points above.

Software and code

Policy information about availability of computer code

Data collection	Data is managed at the H3D Centre with the Dotmatics software.
Data analysis	ZairaChem code is available at https://github.com/ersilia-os/zaira-chem. Extended documentation can be found in the Ersilia Book (ersilia.gitbook.io). ZairaChem benchmarks are reported in https://github.com/ersilia-os/zaira-chem-tdc-benchmark. Code used for analysing data can be found at https://github.com/ersilia-os/h3d-screening-cascade-code. Download links to the fully equipped ZairaChem models are available at: https://github.com/ersilia-os/h3d-screening-cascade-models. A light version of the H3D models is available as a web-based app at h3dscreening.ersilia.io. Code for deployment can be found at https://github.com/ersilia-os/h3d-screening-cascade-app.<br><br>In addition, the following Python packages were used for data analysis: RDKit (v2022.9.5), Chemical Checker Signaturizers (v1.1.10), Grover (v1.1.0), ChemGPT (from Molfeat v0.8.0), Ersilia Model Hub (v0.1.2), Ersilia Embedding (v0.0.1), FLAML (v1.1.2), AutoGluon Tabular (v0.5.2), TabPFN (v0.1.8), MolMap, Keras Tuner (v1.1.3), UMAP Learn (v0.5.3), Matplotlib (v3.6.0), Imbalanced Learn (v0.10.1), Scikit Learn (v0.24), MELLODDY Tuner (v2.1.3).

For manuscripts utilizing custom algorithms or software that are central to the research but not yet described in published literature, software must be made available to editors and reviewers. We strongly encourage code deposition in a community repository (e.g. GitHub). See the Nature Portfolio guidelines for submitting code & software for further information.

Data

Policy information about availability of data

All manuscripts must include a data availability statement. This statement should provide the following information, where applicable:

- Accession codes, unique identifiers, or web links for publicly available datasets
- A description of any restrictions on data availability
- For clinical datasets or third party data, please ensure that the statement adheres to our policy

This work contains AI/ML models build on top of H3D proprietary data. Data is managed at H3D with the Dotmatics software. The identity of the compounds used for training cannot be revealed due to H3D IP constraints. However, all corresponding AI/ML models are publicly available. ChEMBL and PubChem BioAssay were used as additional sources of data (found here: https://github.com/ersilia-os/h3d-screening-cascade-models/tree/main/external_data), as well as the Therapeutics Data Commons (https://tdcommons.ai).

Research involving human participants, their data, or biological material

Policy information about studies with human participants or human data. See also policy information about sex, gender (identity/presentation), and sexual orientation and race, ethnicity and racism.

Reporting on sex and gender	N/A
Reporting on race, ethnicity, or other socially relevant groupings	N/A
Population characteristics	N/A
Recruitment	N/A
Ethics oversight	N/A

Note that full information on the approval of the study protocol must also be provided in the manuscript.

Field-specific reporting

Please select the one below that is the best fit for your research. If you are not sure, read the appropriate sections before making your selection.

☒ Life sciences ☐ Behavioural & social sciences ☐ Ecological, evolutionary & environmental sciences

For a reference copy of the document with all sections, see nature.com/documents/nr-reporting-summary-flat.pdf

Life sciences study design

All studies must disclose on these points even when the disclosure is negative.

Sample size	13,000 small molecules. Sample size was not predetermined: the number of samples for training AI/ML models was determined by the full availability of molecules at the H3D Centre. When less than 100 molecules were available for a specific modeling task, external data was used to complement the dataset, as specified in the manuscript.
Data exclusions	Non-reproducible experiments (relative error >1) were removed from the analysis.
Replication	Data points belong to the internal H3D database, which has high standards of replication and reproducibility, typically in triplicates. All attempts of replication were successful.
Randomization	In the computational cross-validations, stratified splits were performed following standard procedures. 'Experimental groups' (i.e. cross-validation strata) was perform by randomly selecting molecules amongst the 'active' and 'inactives' groups, ensuring active:inactive balance was maintained.
Blinding	N/A. There was no 'blinding' procedure per se. However, we argue that in an AI/ML computational setting, the closest equivalent would be cross-validation, which we performed thoroughly. In particular, we held out repetitively, and randomly in a stratified form, 20% of the data for testing purposes for each of the models, to control for overfitting and have an unbiased estimate of AI/ML models' performance.

Reporting for specific materials, systems and methods

We require information from authors about some types of materials, experimental systems and methods used in many studies. Here, indicate whether each material, system or method listed is relevant to your study. If you are not sure if a list item applies to your research, read the appropriate section before selecting a response.

Materials & experimental systems

n/a	Involved in the study
☒	☐ Antibodies
☒	☐ Eukaryotic cell lines
☒	☐ Palaeontology and archaeology
☒	☐ Animals and other organisms
☒	☐ Clinical data
☒	☐ Dual use research of concern
☒	☐ Plants

Methods

n/a	Involved in the study
☒	☐ ChIP-seq
☒	☐ Flow cytometry
☒	☐ MRI-based neuroimaging
